# Insight evaluation in a Tunisian stabilized outpatients with schizophrenia

**DOI:** 10.1192/j.eurpsy.2024.1576

**Published:** 2024-08-27

**Authors:** S. Ajmi, M. Bouhamed, K. Makni, R. Masmoudi, I. Feki, R. Sallemi, J. Masmoudi

**Affiliations:** Psychiatry A, Hedi Chaker University Hospital, Sfax, Tunisia

## Abstract

**Introduction:**

Schizophrenia is a chronic condition that leads to major socio-professional disintegration and personal suffering. In addition to the classic clinical symptoms, these patients also suffer from poor insight.

**Objectives:**

To assess insight in a population followed up for schizophrenia

**Methods:**

We conducted a cross-sectional and descriptive which concerned the patients followed in the unit of outpatient post-cure consultations of psychiatry ‘A’ at the CHU Hedi Chaker of Sfax. We included 72 stabilized patients diagnosed with schizophrenia according to the DSM criteria 5. For the collection of sociodemographic and clinical data, we used a pre-established sheet. We used the schedule for the Assessment of Insight–Expanded Version(SAI-E) scale to assess clinical insight

**Results:**

The mean age of the patients in our study was 46.83 ± 11.6 years, with a sex ratio (M/F) of 2.

They were single in 48.5%, and unemployed in 69.4%. Their level of education did not exceed primary school at 44.4% and their socio-economic level was low at 63.9%.

In our study, 72.2% of patients had no somatic history and 36.1% had a history of attempted suicide.

Using the SAI-E scale, the mean score was 20.1 with a minimum of 5 and a maximum of 28.

**Conclusions:**

At the end of this evaluation, it is important to emphasize that insight seems to be an important prognostic factor.

**Disclosure of Interest:**

None Declared

